# Differential Defense Responses of Upland and Lowland Switchgrass Cultivars to a Cereal Aphid Pest

**DOI:** 10.3390/ijms21217966

**Published:** 2020-10-27

**Authors:** Lise Pingault, Nathan A. Palmer, Kyle G. Koch, Tiffany Heng-Moss, Jeffrey D. Bradshaw, Javier Seravalli, Paul Twigg, Joe Louis, Gautam Sarath

**Affiliations:** 1 Department of Entomology, University of Nebraska-Lincoln, Lincoln, NE 68583, USA; lise.pingault@unl.edu (L.P.); kyle.koch@unl.edu (K.G.K.); thengmoss2@unl.edu (T.H.-M.); jbradshaw2@unl.edu (J.D.B.); 2 Wheat, Sorghum, and Forage Research Unit, USDA-ARS, Lincoln, NE 68583, USA; nathan.palmer@usda.gov; 3 Redox Biology Center, Department of Biochemistry, University of Nebraska-Lincoln, Lincoln, NE 68588, USA; jseravalli1@unl.edu; 4 Biology Department, University of Nebraska-Kearney, Kearney, NE 68849, USA; twiggp@unk.edu; 5 Department of Biochemistry, University of Nebraska-Lincoln, Lincoln, NE 68583, USA

**Keywords:** switchgrass, *Panicum virgatum*, yellow sugarcane aphid, *Sipha flava*, RNA-seq, transcriptome, gene network, metabolites

## Abstract

Yellow sugarcane aphid (YSA) (*Sipha flava*, Forbes) is a damaging pest on many grasses. Switchgrass (*Panicum virgatum* L.), a perennial C4 grass, has been selected as a bioenergy feedstock because of its perceived resilience to abiotic and biotic stresses. Aphid infestation on switchgrass has the potential to reduce the yields and biomass quantity. Here, the global defense response of switchgrass cultivars Summer and Kanlow to YSA feeding was analyzed by RNA-seq and metabolite analysis at 5, 10, and 15 days after infestation. Genes upregulated by infestation were more common in both cultivars compared to downregulated genes. In total, a higher number of differentially expressed genes (DEGs) were found in the YSA susceptible cultivar (Summer), and fewer DEGs were observed in the YSA resistant cultivar (Kanlow). Interestingly, no downregulated genes were found in common between each time point or between the two switchgrass cultivars. Gene co-expression analysis revealed upregulated genes in Kanlow were associated with functions such as flavonoid, oxidation-response to chemical, or wax composition. Downregulated genes for the cultivar Summer were found in co-expression modules with gene functions related to plant defense mechanisms or cell wall composition. Global analysis of defense networks of the two cultivars uncovered differential mechanisms associated with resistance or susceptibility of switchgrass in response to YSA infestation. Several gene co-expression modules and transcription factors correlated with these differential defense responses. Overall, the YSA-resistant Kanlow plants have an enhanced defense even under aphid uninfested conditions.

## 1. Introduction

Switchgrass (*Panicum virgatum* L.) cultivars originally derived from diverse native populations are genetically distinct from one another and possess significant variation in morphological and physiological traits [1,2]. Consequently, switchgrass cultivars often differ in their responses to biotic and abiotic stress as well [3,4,5,6,7]. Among biotic stressors that have been evaluated, differential responses to insect herbivory have been reported [8,9,10,11]. Plants, especially at the early stages of growth, can be susceptible to aphid infestation, although economic damage to field-grown biomass has not been evaluated. The lowland switchgrass cultivar ‘Kanlow’ had greater resistance to the generalist herbivore fall armyworm (*Spodoptera frugiperda* Smith.), greenbugs (GB; *Schizaphis graminum* Rondani), and the yellow sugarcane aphid (YSA; *Sipha flava* Forbes) compared to the upland cultivar ‘Summer’. The cultivar ‘Summer’ was developed from plants that originated from SE Nebraska USA, and possesses superior winter tolerance with finer tillers and narrow leaves. Whereas the cultivar ‘Kanlow’ was developed from plants collected at a lowland site in Oklahoma, USA [12]. Kanlow plants are taller, with thicker stems and broader leaves. Consequently, Kanlow has superior biomass yields compared to Summer but has poorer winter survival at more norther sites of the USA [13].

Plant defense responses to aphids have been well documented in the literature for several plant–pest interactions, although much current knowledge of these interactions has been derived from model plants, such as Arabidopsis, maize (*Zea mays* L.), and rice (*Oryza sativa* L.) [14,15,16,17,18,19]. In general, work with other plant–aphid systems indicates that early responses to aphid herbivory appears to be largely conserved and follows a pattern of plant perception to mechanical stimuli of aphid movement, cellular reactions triggered in response to aphid probing of cell contents, signaling cascades arising from the influx of Ca^2+^, cellular damage, activation of reactive oxygen intermediates (ROI), and a longer term potentiation arising in response to the loss of carbon assimilation, nutrients and redirection of plant metabolism from growth to defense [17,20,21,22,23,24]. Resistance mechanisms in plants to insect herbivores can arise from several routes and have been classified as antibiosis, where plants contain compounds that affect the biology of the herbivore; antixenosis, where plants contain structures, such as wax, trichomes, spines, that affect herbivore behavior; and tolerance, where plants compensate growth loss of due to herbivory by recalibrating physiological and metabolic processes [21,25,26,27]. Effectively, in host and non-host interactions, plants will mount a defense response to aphid herbivory, and the outcome of these defense responses will determine the longer-term outcomes on plant health. It has been suggested that variations in these longer-term plant responses can differentiate between susceptible and tolerant plants, where susceptible plants are unable to maintain a defense response nor compensate for the loss of sap and nutrients [21,28].

Transcriptional and metabolic outcomes upon infestation with GB have been evaluated in Summer switchgrass plants [29], and those of GB and YSA infestation on Summer and Kanlow switchgrass hybrids [30]. In both studies, similarities and differences were observed in the defense responses to the different aphids. Similarities included upregulation of Ca^2+^ and ROI-related processes, such as mitogen-activated protein (MAP)-kinase signaling, upregulation of genes and enzymes related to redox metabolism, and enhanced levels of pipecolic acid, chlorogenic acid, and other plant defense metabolites. Differences included gene co-expression modules that were enriched only in GB- or in YSA-infested hybrid switchgrass plants. Additionally, GB induced a strong defense response more characteristic of a non-host response, while YSA herbivory was more nuanced, causing a gradual increase in plant defense-related processes over the 15-day time course of the experiment. In both of these studies, several transcription factors (TFs) were identified that were significantly associated with one or more defensive processes [29,30].

Hybrid switchgrass utilized by Koch and colleagues were from a stabilized population derived from crosses of randomly selected Summer (♀) x Kanlow (♂) plants [31]. These plants were able to serve as hosts for both GB and YSA [30], indicating that Kanlow traits regulating GB feeding were suppressed in the hybrids. However, because YSA could infest both Summer and Kanlow plants, it provided a route to investigating different aspects of the defense responses of Kanlow plants to aphid herbivory and simultaneously compare these data with the more permissive host Summer.

## 2. Results

### 2.1. YSA Colonized Both Cultivars with Different Damage Outcomes

**Figure 1:**
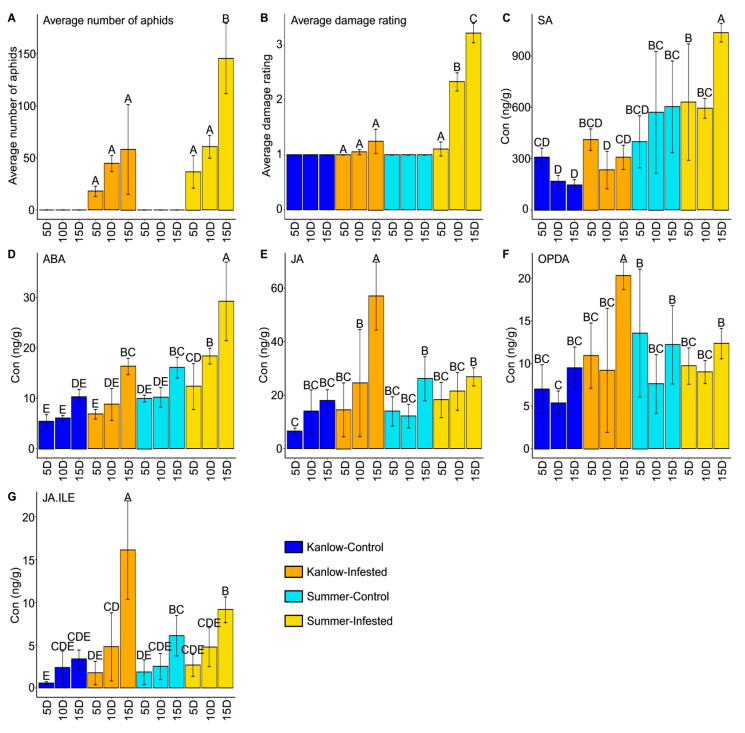
Aphid numbers, damage rating, and phytohormone levels. (**A**) Aphid numbers, (**B**) Plant damage ratings, (**C**) Salicylic acid (SA), (**D**) Abscisic acid (ABA), (**E**) Jasmonic acid (JA), (**F**) 12-Oxo-phytodienoic acid (OPDA), (**G**) Jasmonic acid-isoleucine (JA-Ile). Blue bars are for Kanlow control plants, orange bars are for aphid-infested Kanlow plants, turquoise bars are for Summer control plants, and gold for aphid-infested Summer plants. The letters indicate a significant difference at *p*-value < 0.05, with separation of means using Fisher’s Least Significant Difference (LSD). Error bars represent standard deviation.

No significant difference was observed in aphid numbers for the YSA-infested Kanlow plants at all sampling time points and 5 and 10 Days After Infestation (DAI) for the cultivar Summer. There was a significant increase in aphid numbers between 10 and 15 DAI on the cultivar Summer (Figure 1A; Supplemental Table S1). Infested plants for the cultivar Kanlow did not suffer significant damage across sampling dates (Figure 1B). However, the damage ratings significantly increased for the infested Summer plants at each sampling point, 5 DAI (1.1 ± 0.123), 10 DAI (2.33 ± 0.0165), and 15 DAI (3.22 ± 0.176) (Figure 1B, Supplemental Table S1).

### 2.2. Hormones and Metabolites Were Affected by YSA Infestation

Hormone level variations have been linked to plant defense [32,33]. Here, the quantification of five hormones was performed (Figure 1C–G, Supplemental Table S1). Salicylic acid (SA) levels were significantly greater at 15 DAI for infested Summer plants, whereas no significant differences in SA were found between the control and infested treatments for the cultivar Kanlow (Figure 1C). Abscisic acid (ABA) levels increased over time in infested plants, and levels were significantly higher at 15 DAI in infested Kanlow plants and 10 DAI and 15 DAI for the infested plants of the cultivar Summer compared to their respective controls (Figure 1D). Jasmonic acid (JA), its intermediate 12-oxo-phytodienoic acid (OPDA), and its active form, JA-isoleucine (JA-Ile), were significantly elevated in infested Kanlow plants at 15 DAI compared to the other treatments (Figure 1E–G).

**Figure 2:**
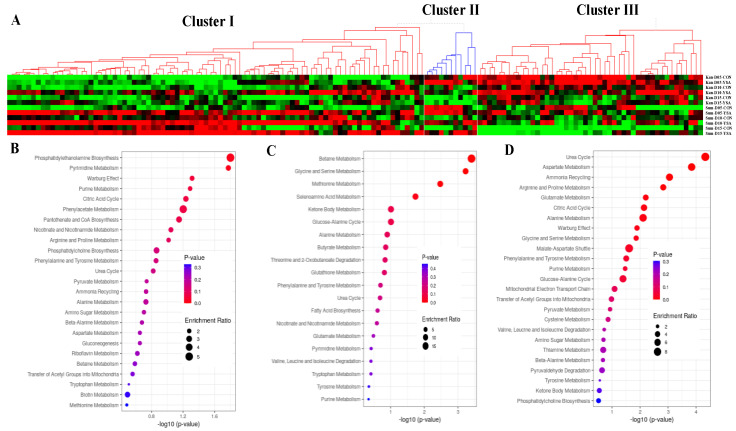
Clustering of the different metabolites enriched in infested and control plants. (**A**) Expression values are given in log_2_(value+1) with z-score normalization. Green below the average. Red above the average. The values are provided in Supplemental Table S1. (**B**) Enrichment of the metabolites for the Cluster I. (**C**) Enrichment of the metabolites for the Cluster II. (**D**) Enrichment of the metabolites for the Cluster III.

The levels of 145 metabolites were measured by LC-MS for each cultivar, time point, and treatment. Hierarchical clustering analysis revealed these metabolites could be organized into 3 clusters, composed of 87 (cluster I), 11 (cluster II), and 47 (cluster III) metabolites, respectively (Figure 2A). Cluster I was related to metabolites upregulated upon infestation in the cultivar Summer, while cluster III was composed of metabolites upregulated in infested Kanlow plants. Metabolites composing cluster II were associated with development time for both cultivars (Figure 2A). Enrichment analysis of the metabolites present in each cluster revealed metabolic pathways common between cluster I and III, such as “Citric Acid Cycle”, “Urea cycle”, “Phenylalanine and Tyrosine Metabolism”, or “Warburg Effect” (Figure 2B,D). Metabolites specifically enriched in cluster I were related to “Nicotianate and Nicotinamide Metabolism”, “Pyrimidine Metabolism”, “Phospatidylethanolamine Biosynthesis”, or “Phenylacetate Metabolism” (Figure 2B). The top five enriched pathways of cluster II were “Betaine Metabolism”, amino acid metabolism pathways (“Glycine and Serine Metabolism”, “Methionine Metabolism”), “Selenoamino Acid Metabolism”, and “Ketone Body Metabolism” (Figure 2C). Metabolites specifically enriched in cluster III were associated with the metabolism of amino acids (e.g., “Arginine and Proline Metabolism”, “Alanine Metabolism”, “Glycine and Serine Metabolism”) (Figure 2D).

### 2.3. Transcriptomic Responses to YSA Infestation

**Figure 3:**
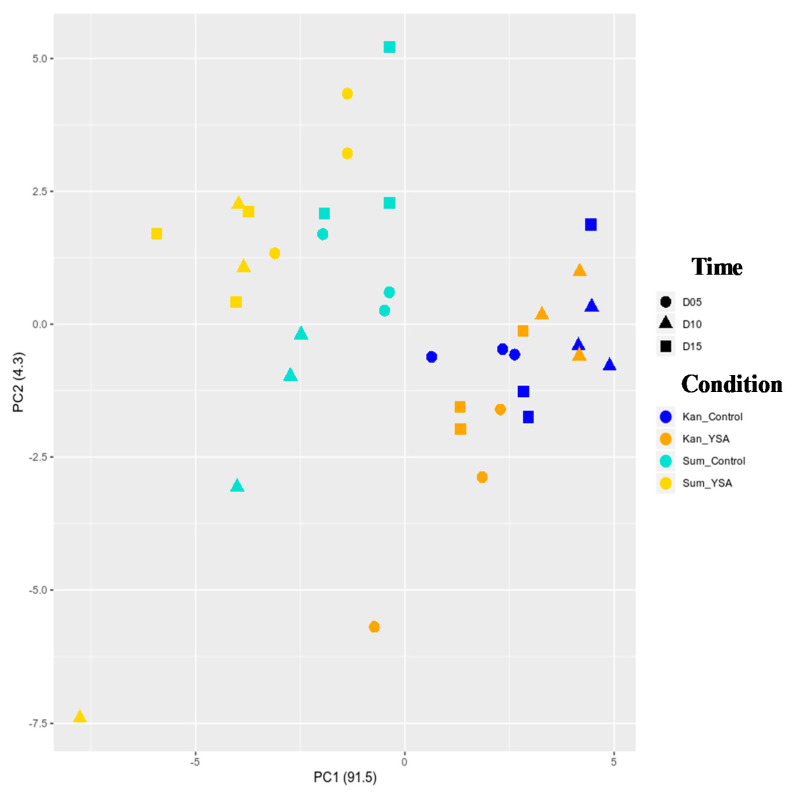
Principal component analysis (PCA) of the 40,657 expressed genes. Conditions are represented with colors (Kanlow control = blue, Kanlow infested = orange, Summer control = turquoise, and Summer infested = yellow) and the time with the shape (5 days = circle, 10 days = triangle, 15 days = square).

A principal component analysis (PCA) of the 40,657 expressed genes was performed (Figure 3, Supplemental Table S2). PC1 accounting for 91.5% of the variance separated the transcriptome by cultivars (PC1, Figure 3). PC2 accounting for 4.3% of the variance indicated a separation between infested versus control plants in Summer but not in Kanlow (Figure 3).

In total, 624 and 4036 genes were differentially expressed in Kanlow and Summer, respectively (Table 1, Supplemental Table S3). For both cultivars, the number of genes upregulated by YSA infestation (log_2_(FC_(YSA/CONTROL)_) ≥ log_2_(3) and *p*-value ≤ 0.05) was higher than the number of genes downregulated by YSA infestation (−log_2_(FC_(YSA/CONTROL)_) ≤ −log_2_(3) and *p*-value ≤ 0.05) (Table 1). When looking at the number of upregulated and downregulated differentially expressed genes (DEGs) for each time point and cultivar (Table 1, Supplemental Figure S1), for cultivar Summer the number of downregulated genes and upregulated genes was approximately similar at 5 DAI (818 and 760 genes, respectively), however at 10 DAI, the number of upregulated genes (574) was much greater compared to the number of downregulated genes (33). At 15 DAI, the number of upregulated and downregulated genes increased greatly compared to 10 DAI (Supplemental Figure S1A). The number of upregulated genes for the cultivar Kanlow varied at each sampling date, and the highest number of upregulated genes was found at 15 DAI (313 genes) (Supplemental Figure S1B). The number of downregulated genes was similar between the three time points (37–57 genes) (Supplemental Figure S1B).

**Figure 4:**
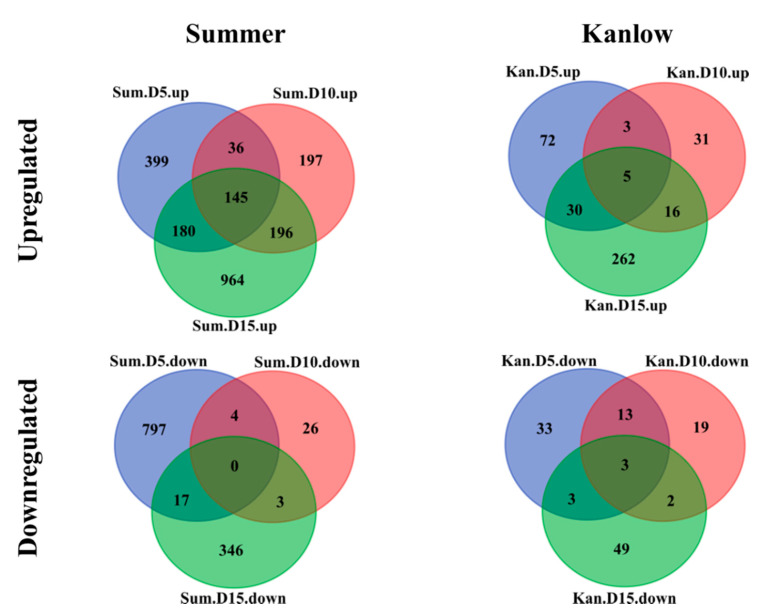
Global changes in differentially expressed genes (DEGs) across time points for each cultivar. Global changes in DEGs are shown as Venn diagrams. Summer DEGs are in the left panel and Kanlow DEGs in the right panel. DEGs are represented for each cultivar between the three time points: 5 Days After Infestation (DAI) (blue), 10 DAI (red), and 15 DAI (green).

In both cultivars, a lower number of DEGs was found at 10 DAI, and the most DEGs were found at 15 DAI. These first results suggested a more rapid change to the transcriptomes in infested Summer plants relative to infested Kanlow plants, although, by 15 DAI, there appeared to be large levels of transcriptional remodeling in both cultivars under YSA infestation. The overlap of DEGs at each time point was next analyzed for each cultivar (Figure 4). Five percent (145 genes) of the total upregulated genes in Summer were shared between the three time points, while 1% (5 genes) for Kanlow were shared between sampling dates (Figure 4, upper panel). Kyoto Encyclopedia of Genes and Genomes (KEGG) pathway enrichment of the 145 genes shared in common across the three sampling dates were related to “metabolic pathways” (21 genes) and “biosynthesis of secondary metabolites” (14 genes), as well as “MAPK signaling pathway” (4 genes), “plant–pathogen interaction” (4 genes), and “plant hormone signal transduction” (4 genes) (Supplemental Table S4). A copy of EGL3 (*Pavir.7KG285720*, *AT1G63650*), a member of the bHLH TF family, was found part of the 145 genes upregulated at all the time points in Summer. In addition, as part of the 145 gene set, two genes responsive to salicylic acid (SA) were also expressed (*Pavir.2KG505500* “receptor lectin kinase”, *Pavir.5KG293200* “pathogenesis-related gene 1”). Among the five genes commonly expressed in the three time points for Kanlow, two have Arabidopsis homologs: *Pavir.7KG285720* (*AT1G63650*), which is a copy of *EGL3* and also upregulated at all three time points in Summer, and *Pavir.8KG224301* (*AT1G78060*) part of the glycosyl hydrolase family protein. The upregulation of Arabidopsis EGL3 homologs in both cultivars under YSA infestation indicates a potential role for this protein in switchgrass defense responses.

**Figure 5:**
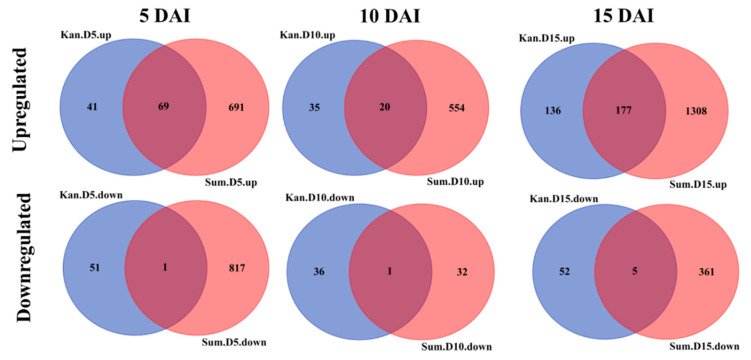
Global changes in DEGs across cultivars for each time point. The Venn Diagrams are represented for each time point between the two cultivars: Kanlow (blue), Summer (red).

Only zero and three genes were downregulated at all three time points for Summer and Kanlow, respectively (Figure 4, lower panel). Among the three genes downregulated in Kanlow, only one has a putative functional characterization related to “cytochrome P450, family 71, subfamily B, polypeptide 13”. The same comparison was performed to identify the overlapping DEGs between the two cultivars at each time point (Figure 5). Between 20 to 177 upregulated genes were induced in common between the two cultivars in response to YSA infestation. These genes represent 36 to 62% of the upregulated genes in Kanlow and 9 to 12% of the upregulated genes for Summer (Figure 5, top panel). At 5 DAI (69 genes upregulated in common) and 15 DAI (177 common genes) have functions related to “flavonoid biosynthesis”, “metabolomics pathways”, “phenylpropanoid biosynthesis”, “biosynthesis of secondary metabolites”, and “plant–pathogen interaction” (Supplemental Table S4). Interestingly, the Arabidopsis EGL3 homologous copy (*Pavir.7KG285720*) has been found upregulated in Summer and Kanlow for the three time points. The low number of genes upregulated in common at 10 DAI (20 genes) did not allow reliable functional enrichment analysis. Similarly, one to five downregulated genes that overlapped between the two genotypes at each time point (Figure 5, lower panel) were not functionally classified.

Overall, the overlap between the three time points for each cultivar indicated a time point specific transcriptomic response. While the overlap of downregulated genes was minimal between the two cultivars for each time point, suggesting cultivar-specific responses to YSA herbivory.

### 2.4. Temporal Gene Expression Varied in Response to YSA Infestation

**Figure 6:**
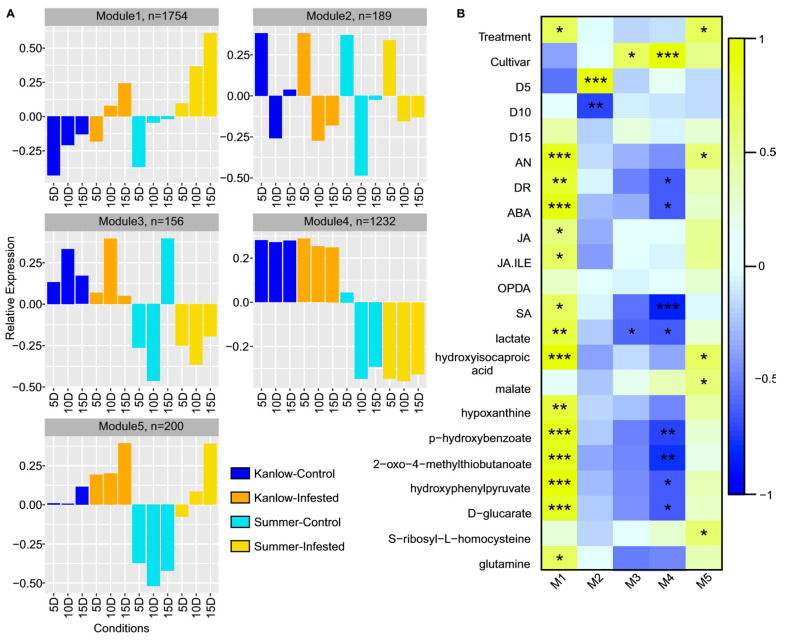
Weighted gene co-expression network analysis (WGCNA) of the 3531 DEGs. (**A**) Expression patterns of genes assigned to five co-expression modules, n indicate the number of DEGs in each WGCNA module. (**B**) WGCNA module and cultivar traits: aphid number (AN), damage rating (DR), hormones, and metabolites relationships. Each column corresponds to a module. Each cell contains the corresponding correlation level (blue–yellow scale) and *p*-value (−Inf < *** < 0.001, 0.001 < ** < 0.001, 0.01 < * < 0.05). For treatment, infested conditions were assigned to 1, and control conditions were assigned to 0. For cultivar, Kanlow conditions were assigned to 1, and Summer conditions were assigned to 0.

In total, 3135 non-redundant DEGs (|log_2_(FC_(YSA/CONTROL)_)| ≥ log_2_(3) and *p*-value ≤ 0.05) were found between the two cultivars and the three time points. A weighted gene co-expression network analysis (WGCNA) was performed and revealed five co-expressed gene modules: Module 1 (M1) through M5. The gene co-expression profiles have shown a variation between time points and cultivars. M1 (1754 genes) and M5 (200 genes) are composed of genes upregulated in Summer and Kanlow under YSA infestation (Figure 6A). M2 (189 genes) contained co-expressed genes upregulated at 5 days in infested and uninfested control plants. Genes present in M3 (156 genes) were upregulated in Kanlow infested and uninfested plants and Summer 15 day uninfested (Figure 6A).

To better interpret the temporal transcriptional activity of each condition/trait, the correlation between the module expression profiles and each trait (i.e., sampling time, treatment, number of aphids, damage rating, hormones, and metabolites) was computed (Figure 6B). M1 and M5 were significantly correlated with YSA infestation for both genotypes (Figure 6B). In addition, M1 co-expressed genes were significantly correlated with the last sampling time point (15 DAI) for aphid numbers and damage ratings (Figure 6B). M2 co-expressed genes were significantly correlated with the genes expressed at 5 DAI (Figure 6B). M3 and M4 co-expressed genes were significantly positively and negatively correlated with the cultivars Kanlow and Summer, respectively (Figure 6B). Altogether, these results showed a temporal-compartmentation of gene transcriptional regulation (up or downregulated) between both cultivars.

Statistical evaluation of metabolite enrichment in samples and their significant association with each module was performed (Supplemental Table S6). A total of 59 metabolites were significantly different in the contrasts analyzed, and their specific association with cultivars and modules varied. Many of these metabolites were enriched in the cultivar ‘Summer’, although 21 metabolites were enriched in both cultivars under infestation at one of the three sampling dates (Supplemental Table S6). Many of these enriched metabolites were significantly associated with M1 and M4 suggesting an underlying commonality to the defense responses between the two cultivars. Three metabolites linked to biosynthetic processes, allantoate, sedoheptulose 1,7-bisphosphate, and 5-phosphoribosyl-1-pyrophosphate, were more depleted in YSA infested Summer plants, potentially indicative of greater stress in the more susceptible cultivar.

Select metabolites and their significant association to modules are indicated in Figure 6B. Notably, several hormones and specific metabolites, such as 2-oxo-4-methylthiobutanoate, p-hydroxybenzoate, and hydroxyphenylpyruvate, arising from amino acid catabolism and required for the biosynthesis of secondary defense compounds, were significantly positively correlated with M1 and negatively correlated with M4. Similarly, hydroxyisocaproic acid, a product of leucine catabolism, and malate were significantly correlated with M1 and M5 and M5, respectively. Both modules were linked to defense responses (Figure 6B).

The compounds 2-oxo-4-methylthiobutanoate, p-hydroxybenzoate, and hydroxyphenylpyruvate are associated with the metabolic pathways “cysteine and methionine pathway”, “phenylpropanoid biosynthesis,” and “phenylalanine biosynthesis”. These metabolite pathways were part of the transcriptomic KEGG enrichment pathways enriched for M1 (Supplemental Table S5). Fourteen genes have a function enriched in “Cysteine and methionine metabolism”. For example, Pavir.6KNG143300 encodes for a “malate dehydrogenase”, Pavir.2KG341300 and Pavir.2NG336090 encode for “S-adenosylmethionine decarboxylase” (Supplemental Table S3). Eighteen genes had a function related to “Phenylalanine metabolism”, among them Pavir.6KG035700 and Pavir.6KG035900 had a function related to “Pyridoxal phosphate (PLP)-dependent transferases superfamily protein”. Finally, fifty-five genes were found with a function enriched in “phenylpropanoid biosynthesis”, including genes with function “peroxidase superfamily protein”. The compound malate, involved in the tricarboxylic acid (TCA) cycle, was found to be related to genes with functions involved in “purine metabolism”, associated with three genes (Pavir.3NG233000, Pavir.7NG434300, and Pavir.8KG109700).

### 2.5. Functional Mechanisms in Response to YSA Infestation

To better understand the co-expression module expression profiles, gene function enrichment was investigated for each module (Supplemental Table S5). M1 (genes upregulated by YSA infestation) was composed of genes associated with plant defense responses, such as response to wounding, phenylpropanoid biosynthesis, and amino acid metabolism. Genes were ranked according to their sum of the expression level for all the conditions (higher to lower), and the analysis of the top 20 highly expressed genes in M1 indicated that these were principally related to vitamin B1 (thiamine) biosynthesis, circadian clock, and flavonoid biosynthesis. Four of the five annotated genes involved in vitamin B1 biosynthesis or response to vitamin B1 were exclusively found in this module: *Pavir.4KG345900* and *Pavir.4NG251200* are homologs to *AT5G54770* annotated as “thiazole biosynthetic enzyme”. *Pavir.9K050400* and *Pavir.9NG091900* are homologs to *AT2G29630* annotated as “thiaminC”.

Correlation between hormone levels and the co-expressed genes of M1 demonstrated a significant positive relationship to SA, ABA, JA, and JA-Ile levels (Figure 6B). However, metabolite enrichment analysis indicated that the thiamine levels were negatively correlated to M1 (Supplemental Table S6). Enrichment analysis of metabolites correlated with M1 was significantly associated with “purine metabolism” and “nitrogen metabolism”. Other genes upregulated in M1 were related to plant defense pathways and included *Pavir.5KG450100* (*AT5G42800*) that encodes a dihydroflavonol reductase, which is involved in the biosynthesis of anthocyanins. Anthocyanins have been identified as a part of toxic chemicals (among terpenoids, alkaloids, phenols, or quinones) produced by plants to either kill or retard the development of insects [34]. *Pavir.8KG261900* (*AT5G13930*) encodes a chalcone synthase (CHS), an enzyme of the flavonoid/isoflavonoid biosynthesis pathway [35].

M2 (genes upregulated at 5 days in both infested and uninfested treatments) had functions associated with nucleic acids’ biosynthesis and ribonucleic activity (Supplemental Table S5). Similar to other modules, genes had functions related to “metabolic pathways” and “biosynthesis of secondary metabolites”. However, several genes were more specifically classified into the following functions: “ribosome biogenesis in eukaryotes”, “pyrimidine metabolism”, “glycine serine and threonine metabolism”, and “starch and sucrose metabolism”, indicating M2 was more linked to growth processes rather than defense-related ones.

### 2.6. Cultivar Specific Functional Mechanisms in Response to YSA Infestation

Genes that were preferentially upregulated under both infested and uninfested conditions in the cultivar Kanlow were functionally related to plant defense (M3, M4, and M5, Figure 6). However, only 156 genes were part of M3, which did not reliably allow enrichment for function. Interestingly, enriched functions specifically found in M4 were associated with “cutin, suberin, and wax biosynthesis” (*Pavir.9NG722200*/*AT3G11980*, *Pavir.6NG369900*/*AT3G44550*, *Pavir.4NG113300*/*AT1G70670,* and *Pavir.2KG092500*/*AT5G41040*) (Supplemental Table S5). The Arabidopsis homologs of these genes encode fatty acid reductase 2 (*AT3G11980*, *FAR2*) and fatty acid reductase 5 (*AT3G44550*, *FAR5*).

Genes composing the M5 (genes upregulated under YSA infestation in both cultivars and downregulated in Summer uninfested control plants) showed functions associated with the regulation of plant defense. KEGG pathways specifically enriched in M5 were related to “sphingolipid metabolism”, “peroxisome”, and “plant–pathogen interaction” (Supplemental Table S5). These gene functions were also reflected in the metabolite pathway enrichment (e.g., “sphingolipid metabolism”, “thiamine metabolism”) (Supplemental Table S6). For the resistant cultivar Kanlow, genes upregulated in both control and infested conditions were associated with basal gene defense-related mechanisms and genes involved in wax/cuticle biosynthesis, potentially providing a prior advantage for aphid resistance.

### 2.7. Transcription Factors’ Role in Transcriptome Response to YSA Infestation

**Figure 7:**
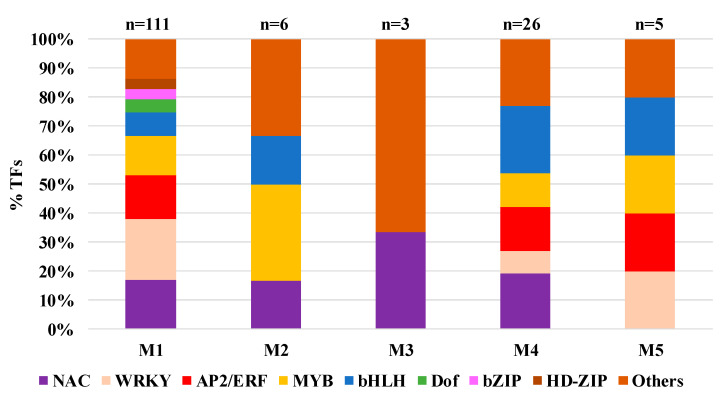
Proportion of (transcription factors) TFs per co-expression module. ‘n’ indicates the number of TFs for each module.

Among the 3531 DEGs, 151 were transcription factors (TFs). The 151 TFs could be organized into 23 families, with 8 TF families containing at least four genes (Supplemental Table S7). The proportion of each of the eight families has been calculated for each co-expression module (Figure 7).

Among the 151 TFs, 111 (73.5%) were part of M1, 26 were part of M4, 6, 3, and 5 TFs were part of M2, M3, and M5, respectively. WRKY (26 TFs), ethylene response factor (ERF) (22 TFs), and MYBs (21 TFs) were present in the M1, M4, and M5. These modules were associated with upregulated genes in infested conditions for both cultivars and Kanlow control condition. NACs (26 TFs) were present in all the modules except M5 (Figure 7, Supplemental Table S7). Interestingly, one TF family (Dof; DNA-binding with one finger), which are a group of plant-specific TFs and mostly expressed in the vascular system [36], were present uniquely in M1 (five TFs).

In addition, MYC2, a TF that belongs to the bHLH family, has been shown to participate in the JA-dependent regulatory process involved in biotic and abiotic stresses [37,38,39,40,41,42]. In the switchgrass genome, 12 homologs of *AtMYC2* (AT1G32640) have been identified (*Pavir.2KG375686*, *Pavir.2KG375706*, *Pavir.2KG375711*, *Pavir.2NG439800*, *Pavir.2NG440500*, *Pavir.2NG440900*, *Pavir.6KG393800*, *Pavir.6KG394000*, *Pavir.6NG052300*, *Pavir.6NG344000*, *Pavir.9KG354713*, *Pavir.9NG353828*). Among these genes, *Pavir.6KG394000* was found to be differentially expressed and part of M4 (Supplemental Table S3, Supplemental Table S7). Another bHLH TF (*Pavir.1KG184800*, *AT4G20970*), expressed in the guard cell in Arabidopsis, has been identified with function related to “defense response to fungus” [43]. Here, *Pavir.1KG184800* was found to be part of M1 and highly expressed in infested Summer (susceptible) plants (Supplemental Table S7).

Among the WRKY TF family, 23 were found in the M1. Among these, five have functions described related to defense (*Pavir.3KG163300* (AtWRKY50), *Pavir.3NG177072* (AtWRKY50), *Pavir.2NG635600* (AtWRKY70), *Pavir.5NG377100* (AtWRKY50), *Pavir.3NG079701* (AtWRKY33)) (Supplemental Tables S3 and S7). WRKY50 is involved in JA inducible defense responses, while WRKY70 functions as an activator of SA-dependent defense genes and a repressor of JA-regulated genes [44]. WRKY70-controlled suppression of JA-signaling is partly executed by NONEXPRESSOR OF PATHOGENESIS-RELATED GENES 1 (NPR1) (not differentially expressed here). WRKY33 has been described as regulating the antagonistic relationship between defense pathways mediating responses to *P. syringae* and necrotrophic fungal pathogens [45]. Other TFs related to plant defense mechanisms were found to be differentially expressed. Among the three copies of ERF4 (homologous to *AT3G15210*) that has been described as a negative regulator of JA-responsive defense gene expression and resistance to a necrotic pathogen, two copies (*Pavir.7KG343600* and *Pavir.7NG406700*) were part of M1, and one (*Pavir.2KG203371*) was found in M4. The Arabidopsis homolog of a calmodulin-binding transcription activator (CAMTA) family TF (*Pavir.9KG253200*/*AT2G22300*) in M3 has been suggested to function in suppressing defense responses through the loss of function mutations, which show enhanced resistance to fungal and bacterial pathogens [46,47].

Three TFs from the FAR1 (FAR-RED IMPAIRED RESPONSE 1) family were found in three modules: M1 (2) and M3 (1) (Supplemental Table S7). FAR1 has been identified in the regulation of chlorophyll biosynthesis in Arabidopsis and also modulating plant immunity [48].

## 3. Discussion

Cultivar specific transcriptomic response to aphid attack has been reported in the literature [49,50]. Similarly, variations in defense mechanisms in response to aphid infestation in switchgrass cultivars have also been identified [8,11,51]. In these earlier evaluations, the upland cultivar Summer was identified as being susceptible to YSA, whereas the lowland cultivar Kanlow was identified as resistant to YSA [11]. Additionally, another important cereal aphid pest, GB, could not utilize Kanlow as a host but could colonize cultivar Summer and a hybrid derived from Summer x Kanlow plants [8]. These earlier findings suggested that Kanlow might possess broad resistance to aphids, and GB and YSA, in particular. Here, we combined global transcriptomic and metabolomic approaches to investigate defense-regulated pathways important for switchgrass response to YSA attack using Kanlow and Summer plants. Kanlow and Summer plants are important sources of genetics used for breeding to improved switchgrass cultivars adapted to the US Midwest [52,53,54]. Similar approaches have been used by other researchers to understand plant–aphid interactions [55,56,57,58,59,60,61]. As examples, Zhang et al. [56] found that GB infestation of wheat increases endogenous reactive oxygen species (ROS) production compared to the non-phytotoxic aphid *Sitobion avenae*, suggesting a similarity in responses observed in Summer and hybrid switchgrass infested with GB or YSA [29,30]. Phloem-resident mechanisms (other than callose) can also impact aphid and virus responses [62], although it is unclear if such mechanisms are resident in Kanlow plants [4].

### 3.1. Commonalities in Defense Response between Both Cultivars

Many commonalities in the defense responses of plants to a variety of insect pests have been documented in the literature [32,63,64,65]. Genotypes (cultivars) within a single species could have differential resistance to a given aphid but could have similarities in their basal and/or even longer-term defense responses [66,67]. Data from this current report reinforce and extend these earlier findings to the biofuel crop, switchgrass. At 15 DAI, the 117 commonly upregulated genes were associated with functions related to the biosynthesis of secondary metabolites and amino acids. These results were confirmed at the metabolite level for several compounds, such as phenylalanine and tyrosine. Diversion of primary metabolites and metabolism to secondary metabolites and metabolism has been identified as a common thread in plant defense responses to biotic stress [68,69].

### 3.2. Rate in the Change of Plant Damage and Gene Expression/Metabolomics Variation over the Time

The temporal regulation of the transcriptome was impacted by YSA herbivory, but the two switchgrass cultivars did not respond defensively to this attack with the same timing or the same intensity. Indeed, the upregulated genes were in greater number at 5 DAI in Summer, while the greatest number of upregulated genes was found at 15 DAI for the cultivar Kanlow.

Basic defense functions also did not have the same temporal transcription in both cultivars. These functions were related to genes involved in multiple related pathways, namely flavonoid and phenylpropanoid biosynthesis, phenylalanine metabolism, or diterpenoid metabolism. These pathways have been related to the chemical and mechanical response to aphid feeding through the action of salivary effectors or elicitors [57,70,71,72,73]. The flavonoid pathway was specifically induced by YSA and not by GB in the hybrid derived from Summer x Kanlow plants [30]. Similar changes in flavonoid metabolism have been reported for the soybean–soybean aphid (*Aphis glycines*) interactions [72]. Terpenoid synthesis has been shown to be a defense mechanism for plants in response to insect attack in switchgrass [5], and volatile terpenoids can also act as an attractant for natural predators of insect herbivores [74,75]. Here, the results indicate that similar basal defense mechanisms were activated in both cultivars. However, looking at the expression level of the genes, these mechanisms were highly expressed in the Summer (susceptible) cultivar (M1, Figure 6A). Plant damage increased over time in the Summer cultivar and was correlated with aphid numbers. A similar observation has been made for the hybrid switchgrass in response to YSA infestation [30]. Indeed, the YSA numbers and plant damage did not increase significantly on the cultivar Kanlow over the time course of the experiment. These results could explain the muted transcriptomic response associated with plant defense mechanisms in Kanlow compared to Summer under YSA pressure.

Gene expression in infested Summer plants over the three sampling points was correlated with hormone levels. For example, genes involved in the biosynthesis of Vitamin B1 were upregulated at all three sampling dates in the Summer cultivar and their expression increased over time. In Arabidopsis, biosynthesis of Vitamin B1 is regulated through the SA signaling pathway [76]. Vitamin B1 is essential for proper cellular functions and plays an important role in carbohydrate catabolism, NADPH and ATP biosynthesis, and the formation of nucleic acids. Vitamin B1 has also been described as needed for plant adaptation to biotic and abiotic stresses [76,77,78,79], and its synthesis is activated during plant adaptation to persistent abiotic stress conditions. Vitamin B1 has been identified as inducing systemic acquired resistance (SAR) [76]. In rice, exogenous thiamin induced a transient expression of pathogenesis-related genes and upregulation of protein kinase C activity. In Arabidopsis, thiamine induces systemic acquired resistance through the SA and calcium-related signaling pathways. Here, thiamine associated genes were a significant part of the M1 (Summer defense upregulated genes) and correlated with SA levels. Chalcone synthase (CHS) was also part of M1. In soybean, CHS was activated by JA signaling [80]. Genes upregulated in M1 were also positively correlated with JA, which could explain CHS transcription. Increased SA levels were associated with the induction of the flavonoid pathway genes and flavonoid content in YSA, but not GB-infested hybrid switchgrass [30]. The current results corroborate these earlier findings. Anthocyanin accumulation has been linked to YSA herbivory in *Sorghum halepense* [81] and could be a common defensive response in grasses infested with YSA.

Phytohormone analysis documented a significant increase in SA and ABA levels at 15 DAI in Summer. However, SA levels did not change significantly in Kanlow, indicating that the SA pathway was not activated in the cultivar Kanlow in response to YSA attack. NPR proteins (NPR1, NPR3, and NPR4) have been identified in providing a role in plant defense response via SA-pathway [82]. Here, one gene (*Pavir.9KG062457*) that encodes for NPR4 was downregulated at 15 DAI in Summer. It has been reported that NPR4 was downregulated under a high SA level [83], which is consistent with our results. Conversely, JA and JA-Ile were induced at 15 DAI for Kanlow, which is consistent with many previous analyses showing that JA is highly involved in the activation of plant defense related to aphid attacks [84]. The level of ABA increased at 15 DAI in Kanlow to approximately similar levels seen at 10 DAI for infested Summer plants. Transcriptomic studies on Arabidopsis infested with green peach aphids (*Myzus persicae*) indicate that ABA-induced genes were beneficial for aphids [85].

### 3.3. Evidence for Differential Priming of Aphid Defense Mechanisms

It has been demonstrated that Kanlow has a high level of antibiosis resistance mechanisms in response to YSA attack [11]. Our RNA-seq analysis showed a low variation/change of the transcriptomes between control and YSA-infested Kanlow plants. Conversely, the YSA susceptible Summer showed large transcriptomic plasticity between control and infested conditions. Previously, the transcriptomic response of resistant and susceptible sorghum genotypes to sugarcane aphid infestation, suggesting that the resistant genotype had higher numbers of DEGs compared to the susceptible genotype [49]. Similar results were obtained in soybean lines infested with soybean aphids [86]. Collectively, these results suggested that elevated transcription of plant defense mechanisms could basally be higher in Kanlow relative to Summer, consistent with data reported for Kanlow and Summer plants raised under optimal greenhouse conditions [87]. A group of co-expressed genes comprising M4 were upregulated in YSA-uninfested and infested plants of Kanlow and downregulated in Summer. Several of these genes were annotated as having functions related to chitin binding or fatty-acid reductases. *FAR5* has been shown to generate fatty acids found in wound-induced leaf tissue in Arabidopsis [88]. *AT5G41040* encodes a feruloyl-CoA transferase required for suberin synthesis [89]. *Pavir.8KG305700*/*AT3G04720* encodes a protein similar to the antifungal chitin-binding protein. The transcription of this gene increased in response to ethylene [90]. Having these genes highly expressed in uninfested plants could confer Kanlow a greater resistance to aphids, consistent with earlier published studies [8,11,29].

Many TF families have been identified as playing a role in response to insect herbivores in other plants [9,56,91,92,93]. Here, two members of the WRKY family (WRKY50 and WRKY70) were upregulated after aphid attacks for both cultivars. These two TFs have been identified as upregulated at 15 DAI in hybrid switchgrass (Summer x Kanlow) as well [30]. Genes encoding another TF family, Dof, were present in M1 (upregulated genes in both cultivars after YSA attack). Several members of this Dof TF family were upregulated in response to abiotic and biotic stresses [94,95,96]. In cucumber, cis-element analysis of Dof was identified and associated with hormone signaling (e.g., ethylene), among other signals [97].

Epicuticular wax composition has been identified as playing a role in increasing or decreasing the capacity of aphids to feed on the plant [98,99,100]. Among the components of epicuticular waxes are triterpenoids, free fatty acids alcohols, or free fatty acids, some of which could conceivably impact aphid feeding. Here genes that were part of M4 (upregulated in Kanlow for both uninfested and infested conditions compare to Summer) were enriched for proteins required for cutin, suberin, and wax biosynthesis. Among the genes of M4, *Pavir.6KG267500* has been recently identified as part of one of the quantitative trait loci (QTLs) associated with surface wax in switchgrass [101]. This gene is a homolog of a wax associated barley gene: CERQ lipase/carboxyl transferase, which is part of the beta-diketone synthase polyketide pathway [101]. How the genes related to epicuticular waxes might impact YSA herbivory of Kanlow plants is unclear at present. Indeed, EPG analysis of YSA feeding did not provide an obvious hypothesis for surface or phloem-based factors contributing to resistance mechanisms to YSA in Kanlow plants [4]. Our results would support multiple mechanisms that could underlie Kanlow resistance to cereal aphids.

## 4. Materials and Methods

### 4.1. Plant Growth Conditions and Treatments

Seeds of the cultivars Summer and Kanlow were grown in individual SC-10 Super Cell Single Cell Cone-containers (Stuewe & Sons, Inc., Corvallis, OR). Plants were grown in a greenhouse, as previously described [4,8,11,29,102]. The plants were arranged in 3 × 4 × 3 factorial design, with 3 replicates consisting of 6 individual plants (genotypes) each of control and infested treatments, 2 cultivars, and 3 sampling time points at 5, 10, and 15 days after infestation (DAI). For infestations, 10 apterous YSA were placed on individual plants at the V3 stage at day 0. Individual infested and control (uninfested) plants were caged with tubular plastic cages with vents covered with organdy fabric to confine YSA on the plants. Before sampling, aphids were removed with a fine-tipped brush and counted. Visual damage ratings were assigned to each individual plant on a 1 to 5 scale, where 1 represented minimal or no damage, and 5 was extensive damage or dead plant [8,11,102]. Whole plants were collected and flash-frozen in liquid nitrogen, ground to a fine powder, and stored at −80 °C until utilized. All collections were performed around 10 am to minimize effects arising from diurnal cycles.

### 4.2. RNA Extraction and Sequencing

Three biological replicates (consisting of 6 individual plants each) were processed for each time point and treatment. A total of 36 samples were used for RNA extractions. RNA was isolated, and 100-bp single-end (SE) sequencing was done, as previously described [29,30,103]. The transcriptomics dataset is available Bioproject: PRJNA657564.

### 4.3. RNA-Seq Analysis

Libraries QC was performed with FastQC. Reads were trimmed with bbduk (part of BBTools; https://jgi.doe.gov/data-and-tools/bbtools/) using the following parameters: ftl = 11 k = 13 ktrim = r ftm = 5 useshortkmers = t mink = 5 qtrim = t trimq = 10 minlength = 30. Then, cleaned reads were mapped on the switchgrass genome (version 5.1, phytozome.jgi.doe.gov) [104] using STAR [105] and gene counts were tabulated using featureCounts [106]. The RNA-seq datasets used in this article are available in the SRA repository at the accession number PRJNA657564.

Differentially expressed genes (DEGs) were identified with DESeq2 [107] and defined by having |log_2_(fold-change)| ≥ log_2_(3) and *p*-value ≤ 5% between the infested time points and its related control. Co-expression modules were identified by the weighted gene co-expression network (WGCNA) [108].

### 4.4. Gene Ontology (GO) Analysis

The GOBU package was used for enrichment calculations [109]. The full set of switchgrass gene annotation was used as the reference comparison set against each DEGs list. *p*-values were calculated using Fisher’s exact test, and they were corrected for multiple testing with the false discovery rate (FDR) method with the R package ‘*p*-adjust’. Transcription factors (TFs) classes were identified using the Family Assignment Rules used by the PlantTFDB v5.0 (http://planttfdb.gao-lab.org).

### 4.5. Plant Hormone and Metabolite Analyses

Plant hormones were extracted from 50 mg of ground tissue in methanol/acetonitrile (1:1 v/v) and analyzed by LC-MS/MS [110,111]. Polar metabolites were extracted from 50 mg of ground tissue in 80% methanol, and targeted metabolites were quantitated by multiple reaction monitoring (LC-MRM-MS) analysis [112,113]. Metabolites were assigned to pathways using the Kyoto Encyclopedia of Genes and Genomes (KEGG) [114]. Metabolite enrichment was performed with MetaboAnalyst (https://www.metaboanalyst.ca/). The pairwise comparison between the infested condition and their corresponding control was performed with R and the function pairwise *t*-test.

## 5. Conclusions

In this study, we provide an overview of two distinct omics based regulatory pathways in response to YSA feeding susceptible (Summer) and resistant (Kanlow) switchgrass cultivars. With time, the susceptible cultivar Summer had high plant damage ratings and aphid numbers. In response, a cascade of defense response mechanisms was activated as deduced at the transcriptomic and metabolomic levels. Conversely, the resistant cultivar Kanlow had a low level of damage due to YSA infestation, resulting in a more muted response at the transcriptomic and metabolomic levels. However, Kanlow appeared to possess an elevation of defense-related responses in the uninfested state, potentially contributing to its observed resistance to YSA herbivory.

## Figures and Tables

**Table 1:** Number of differentially expressed genes (DEGs) under different conditions.

	Conditions	Up	Down	Gene Number
**Kanlow**	5 DAI	110	52	162
	10 DAI	55	37	92
	15 DAI	313	57	370
**Summer**	5 DAI	760	818	1578
	10 DAI	574	33	607
	15 DAI	1485	366	1851

## Supplementary Materials

Supplementary Materials can be found at https://www.mdpi.com/1422-0067/21/21/7966/s1. Supplemental Figure S1: Global changes in DEGs. Number of DEGs upregulated (blue) or downregulated (orange) for each switchgrass genotype at 5 DAI, 10 DAI, and 15 DAI of YSA. (A) Summer and (B) Kanlow. Supplemental Table S1: Damage rating and hormone levels for each condition. Supplemental Table S2: RNA-seq data raw count. Supplemental Table S3: Contrast summary. Supplemental Table S4: KEGG pathway enrichment. Supplemental Table S5: Number of DEGs per KEGG pathway, biological process, and molecular function for each co-expression module (KEGG pathways specifically found in a specific module are highlighted with different colors). Supplemental Table S6: Correlation level between significant metabolites and WGCNA modules. Supplemental Table S7: Transcription factors DEGs.
