# Supplementary material for: Differential Defense Responses of Upland and Lowland Switchgrass Cultivars to a Cereal Aphid Pest
Source: Int J Mol Sci. 2020 Oct 27;21(21):7966. doi: 10.3390/ijms21217966 (PMC7672581; doi:10.3390/ijms21217966)
Supplement: Supplementary file 1 [file ijms-21-07966-s001.zip › Supplemental_figure.pptx]

## Slide 1
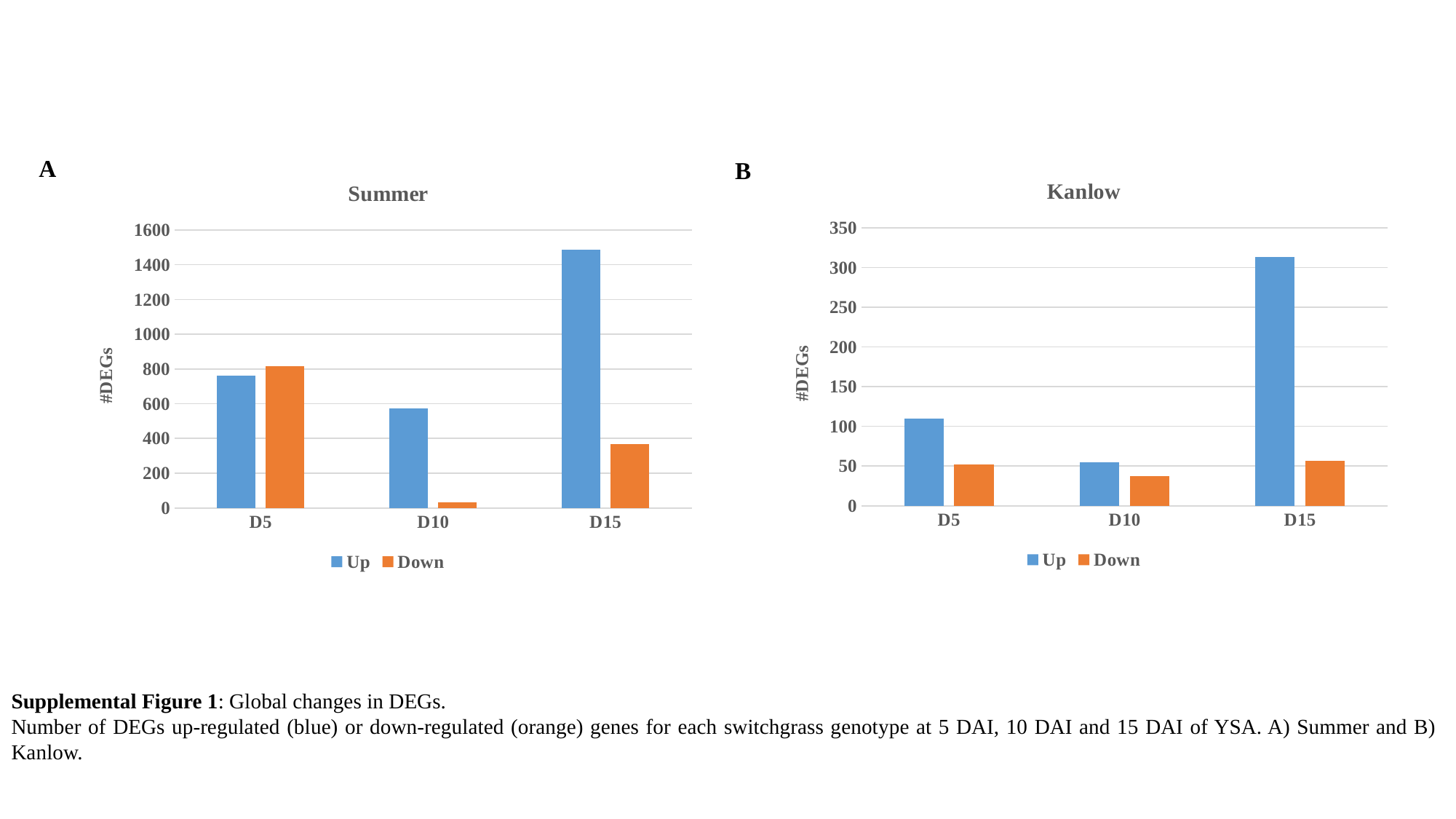

A
B
### Chart: Kanlow
| Category | Up | Down |
|---|---|---|
| D5 | 110.0 | 52.0 |
| D10 | 55.0 | 37.0 |
| D15 | 313.0 | 57.0 |
### Chart: Summer
| Category | | |
|---|---|---|
| D5 | 760.0 | 818.0 |
| D10 | 574.0 | 33.0 |
| D15 | 1485.0 | 366.0 |Supplemental Figure 1: Global changes in DEGs.
Number of DEGs up-regulated (blue) or down-regulated (orange) genes for each switchgrass genotype at 5 DAI, 10 DAI and 15 DAI of YSA. A) Summer and B) Kanlow.
